# Local and International Implications of Schistosomiasis Acquired in Corsica, France

**DOI:** 10.3201/eid2110.150881

**Published:** 2015-10

**Authors:** Philippe Gautret, Frank P. Mockenhaupt, Frank von Sonnenburg, Camilla Rothe, Michael Libman, Kristina Van De Winkel, Emmanuel Bottieau, Martin P. Grobusch, Davidson H. Hamer, Douglas H. Esposito, Philippe Parola, Patricia Schlagenhauf, for the GeoSentinel Surveillance Network

**Affiliations:** Author affiliations: Institut Hospitalo-Universitaire Méditerranée Infection, Marseille, France (P. Gautret, P. Parola);; Aix Marseille Université, Marseille (P. Gautret, P. Parola);; Charité–Universitätsmedizin Berlin, Berlin, Germany (F.P. Mockenhaupt);; University of Munich, Munich, Germany (F. von Sonnenburg);; University Clinic Hamburg–Eppendorf, Hamburg, Germany (C. Rothe);; McGill University, Montreal, Quebec, Canada (M. Libman);; University Hospital, Ghent, Belgium (K. Van De Winkel);; Institute of Tropical Medicine, Antwerp, Belgium (E. Bottieau);; University of Amsterdam, Amsterdam, the Netherlands (M.P. Grobusch);; Boston Medical Center, Boston, Massachusetts, USA (D.H. Hamer);; Centers for Disease Control and Prevention, Atlanta, Georgia, USA (D.H. Esposito);; University of Zürich Centre for Travel Medicine, Zurich, Switzerland (P. Schlagenhauf)

**Keywords:** schistosomiasis, Corsica, France, travelers, parasites, freshwater rivers, Schistosoma haematobium, trematode, snail, intermediate host, host, urinary schistosomiasis, surveillance, epidemiology, Bulinus truncatus snail, One Health, outbreak

## Abstract

We report 11 cases of schistosomiasis in international travelers who had bathed in rivers in Corsica, France, during 2012–2014. The infections were diagnosed in 2014 and reported to the GeoSentinel Surveillance Network and European Travel Medicine Network. Travelers can be sentinels for emerging infections; thus, this situation warrants a concerted human and veterinary epidemiologic response.

In 2014, reports were received of several cases of *Schistosoma haematobium* trematode infection acquired in Corsica, a Mediterranean French island. The first patient was a child from Germany who had traveled to southern Corsica in August 2013 and had no other known exposures. Medical examination showed that the child had gross hematuria; he received a diagnosis of urinary schistosomiasis (*1*). Serologic test results were positive for 4 of 5 asymptomatic family members who had also traveled to Corsica and bathed in the Cavu River, near Porto-Vecchio. Eleven additional cases of urinary schistosomiasis were reported among mainland French tourists who bathed in the Cavu River during August 2011–August 2013 (*2*–*4*). All cases were identified during the chronic phase of the disease.

Following a national screening campaign in France, ≈100 additional schistosomiasis cases were described over 2 years among Corsica residents and visitors from other parts of France. In addition, a competent intermediate host snail, *Bulinus truncatus*, was identified in rivers in Corsica (Technical Appendix Table 1). To elucidate the epidemiologic characteristics of schistosomiasis in Corsica, we determined the number of and clinical findings for urinary schistosomiasis cases among international travelers to Corsica.

## The Patients

To find cases, we searched the GeoSentinel Surveillance Network database (*5*) for urinary schistosomiasis diagnoses for international travelers with freshwater (river) exposure in Corsica during 1996 through March 2015. We also informally extended the search to members of the European Travel Medicine Network (EuroTravNet, http://www.istm.org/eurotravnet). GeoSentinel’s data-collection protocol is for public health surveillance, so human subjects’ review board clearance was not needed. According to local institutional review boards at sites in Berlin and Munich, Germany, our use of supplemental data not available in the GeoSentinel database conforms to the human subject protection guidelines at these sites. Individual patient consent was obtained at sites in Hamburg, Germany; Ghent, Belgium; and Montreal, Quebec, Canada.

We identified 11 records with diagnoses of schistosomiasis acquired in Corsica during 1996–March 2015 (Table). Patients resided in Germany, Belgium, or Canada and had traveled to Corsica during 2012–2014; some had also traveled to Corsica before 2012. No patients reported other exposure to freshwater in any other country where schistosomiasis is known to be endemic. All 11 patients reported bathing in rivers in Corsica: 7 persons (6 from Germany, 1 from Canada) bathed in the Cavu River; a familial cluster of 3 persons from Berlin bathed in the Gaglioli, Solenzara, and Restonica Rivers; and a Belgian tourist bathed in the Osu River.

**Table:** Characteristics for 11 international travelers with urinary schistosomiasis diagnosed in 2014 and acquired in Corsica, France*

Patient age, y/sex	Reporting site	Travel to Corsica		First clinic visit, 2014	Eosinophils, cells/μL	Serologic test result		Eggs in urine	Infection status†
		2014	Before 2014			First-line (antibody titer, IU)	Second-line		
7/M	Berlin, Germany	None	2013	Jun 5	1,415	Pos (26.0)‡	ELISA and IHA Neg§	Neg	Probable
52/M	Berlin	None	2012	Jul 18	192	Borderline (11.0)‡	ELISA and IHA Neg§	Neg	Suspected
29/F	Ghent, Belgium	None	2013	Aug 29	560	Pos (1.1)¶	IHA Neg#	Neg	Probable
17/F	Hamburg, Germany	July–August	None	Sep 8	45	Weak Pos**	IIFT Neg	NT	Probable
11/M	Munich, Germany	None	2013	Sep 22	770	Pos††	IIFT Pos	Pos	Confirmed
35/F‡‡	Berlin	July	2011–2013	Sep 23	202	Pos (17.0)‡	ELISA and IHA Neg§	Neg	Probable
35/M‡‡	Berlin	July-August	2012–2013	Oct 7	359	Pos (29.0)‡	ELISA and IHA Neg§	Neg	Probable
11/M‡‡	Berlin	July–August	2012–2013	Oct 7	152	Borderline (9.0)‡	ELISA and IHA Neg§	Neg	Suspected
41/F	Munich	May–June	2007, 2009–2011	Oct 10	344	Pos††	IIFT Pos	Neg	Confirmed
45/F	Montreal, Quebec, Canada	July	None	Dec 5	0	Pos (0.37)§§	ND	Neg	Suspected
6/F	Berlin	None	2011–2012	Dec 22	950	Pos (32.0)‡	ELISA and IHA Neg§	Neg	Probable

*All patients were detected through a search of the GeoSentinel Surveillance Network (*5*). The patient from Montreal had microscopic hematuria; none of the other patients showed signs or symptoms of disease, and their infections were found during screening. IHA, indirect hemagglutination assay; IIFT, indirect immunofluorescence test; Neg, negative; ND, not done; NT, not tested; Pos, positive. †Suspected, borderline result from 1 serologic testing method; probable, positive result from 1 serologic testing method; confirmed, positive result from 2 serologic testing methods and/or parasite eggs in urine. ‡As determined by using a *Schistosoma mansoni* IgG ELISA (DRG Diagnostics, Marburg, Germany); negative, <9; borderline, 9–11; positive, >11. §As determined by using an *S. mansoni* adult or egg IgG ELISA; IHA, Cellognost Schistosomiasis H (Siemens, Erlangen, Germany). ¶As determined by using an in-house *S. mansoni* IgG ELISA using egg antigen extract mixed with *S. mansoni* adult worm extract imported from Egypt (positive at an optical density >1). #As determined by using an in-house IHA with an *S. mansoni* adult worm extract (Fumouze SA, Levallois-Perret, France), with titration and cut-off set at 1/80 (positive at >1/160). **As determined by using an in-house *S. mansoni* cercariae IgG ELISA. ††As determined by using an in-house *S. mansoni* IgG ELISA. ‡‡Familial cluster. §§As determined by using an in-house *S. mansoni*–*S. haematobium* combined IgG ELISA (negative, <0.3; borderline, 0.3–0.35; positive, >0.35).

All infections were in asymptomatic persons who sought screening in 2014 after seeing/hearing public health warnings regarding the risk for acquiring schistosomiasis after freshwater exposure in Corsica. Four cases were in children <15 years of age; 1 was in a 17-year-old girl. Two recent infections were acquired after exposure in July or August 2014.

Eosinophilia was recorded for 4 patients (Table). Most diagnoses relied on serologic testing, including the diagnoses for 2 patients who were suspected to have schistosomiasis because of repeated borderline seropositive test results or borderline results plus being part of a familial cluster. Parasite eggs were identified in only 1 patient.

## Conclusions

We document 11 cases of schistosomiasis in international travelers who had freshwater river exposure in Corsica during 2012–2014. Of note, 4 of the persons did not report bathing in the Cavu River (the source of all cases of *S. haematobium* trematode infection among French patients so far), but they did bathe in other Corsica rivers.

Corsica has a population of 316,000 persons, but in 2012, the island was visited by 2.7 million tourists, primarily from mainland France, followed by Italy, Belgium, Germany, and Switzerland (Technical Appendix Table 2). Local data from the Porto-Vecchio community (≈10,000 inhabitants) confirm the predominance of French nationals among the ≈100,000 tourists who visited the area in 2011; much smaller numbers of tourists visited from other parts of Europe and North America (Technical Appendix Table 2). Because tourists outnumber residents, it is not surprising that most persons who acquired urinary schistosomiasis in Corsica were tourists (Technical Appendix Figure). French travelers are not represented in GeoSentinel data because travel across an international border is required for inclusion in the database (*5*). The predominance of German travelers in our cohort may reflect the high proportion of Germans among international travelers to Corsica and the strong representation of German travelers in the GeoSentinel database.

Our report has limitations. All patients identified through the Surveillance Network were asymptomatic at the time of diagnosis. Only 1 traveler had parasitologic proof of infection. In these patients, a diagnosis of schistosomiasis was made on the basis of only 1 positive serologic test result; in some cases, the results were borderline or weakly positive. Serum samples were examined by using in-house or commercial assays and were not tested side by side in 1 reference laboratory or confirmed by Western blot. We cannot completely exclude that the case definition in our study generated false-positive cases; diagnosis of schistosomiasis in a setting where the disease is not endemic is extremely challenging (*6*). Symptoms of acute schistosomiasis (corresponding to larval migration) may be absent (as in our cases) or nonspecific, but chronic infection (presence of adult worms) due to *S. haematobium* is symptomatic in ≈66% of cases with detectable egg excretion (often of light intensity however) (*7*). In acute and chronic infections in travelers, sensitivity of egg detection is notoriously poor, and serologic test performance is far from optimal.

Schistosomiasis has never been established in Europe. However, sporadic autochthonous cases of human urinary schistosomiasis were reported in Greece, Cyprus, Spain, and Portugal in the 1920s (*8*); the last cases were reported in Portugal in 1965 (*9*). Autochthonous transmission of urinary schistosomiasis to humans has only recently been described in France (*10*). The intermediate host snail, *B. truncatus*, is widely distributed in Africa, the Middle East, and the Mediterranean Basin as far north as Portugal, Spain, Sardinia, and Corsica (*11*). Because the intermediate host is present and climatic conditions are suitable, the risk for autochthonous transmission of *S. haematobium* in the region of Porto-Vecchio was predicted as early as 1928 (*12*). Animal schistosomiasis caused by *S. bovis* was described in cattle in Corsica in 1929; *B. truncatus* snails were identified as the intermediate host. The last cases of animal schistosomiasis in Corsica were documented in 1966 (*13*). The discovery of human cases of schistosomiasis proves that a human–*Bulinus* parasitic cycle exists in Corsica and suggests that a cattle–*Bulinus* cycle may also exist (*13*). Furthermore, hybridization between schistosome species can occur, specifically hybridization of *S. bovis* and *S. haematobium*, as described in Senegal and elsewhere (*14*). Hybridization results in heterosis, thereby producing offspring that have higher fecundity, faster maturation, and a wider intermediate host spectrum.

The situation in Corsica is of significance for One Health medicine and disease epidemiology and, thus, requires a concerted public health and veterinary epidemiologic response. Because a competent intermediate host is present and schistosomes can be imported by migrants and travelers returning (primarily) from sub-Saharan Africa, autochthonous foci of schistosomiasis could become established throughout susceptible Mediterranean areas in southern Europe. The latest data from EuroTravNet indicate that among the >32,000 travelers who returned home ill during 2008–2012, schistosomiasis ranked twelth among all diagnoses (*15*), and travelers were infected almost exclusively in Africa; none were infected in Europe. We hypothesize that the schistosomiasis outbreak in Corsica began with importation of *S. haematobium* trematodes and subsequent establishment of an autochthonous transmission cycle.

Technical Appendix Public health measures in France regarding the emergence of schistosomiasis in Corsica, tourism statistics for Corsica, and tracking of schistosomiasis cases linked to Corsica.
