# Supplementary material for: Local and International Implications of Schistosomiasis Acquired in Corsica, France
Source: Emerg Infect Dis. 2015 Oct;21(10):1865–8. doi: 10.3201/eid2110.150881 (PMC4593456; doi:10.3201/eid2110.150881)
Supplement: Technical Appendix — Public health measures in France regarding the emergence of schistosomiasis in Corsica, tourism statistics for Corsica, and tracking of schistosomiasis cases linked to Corsica. [file 15-0881-Techapp-s1.pdf]

Article DOI: http://dx.doi.org/10.3201/eid2110.150881

Local and International Implications of Schistosomiasis Acquired in Corsica, France

Technical Appendix

Technical Appendix Table 1. Summary public health measures in France regarding the emergence of schistosomiasis in Corsica*

Date initiated–date ended	Measure
June 2014	Prohibition bathing in or having other contact with water from the Cavu River and urinating in certain Corsican rivers to disrupt the infection cycle
June–November 26, 2014	Serologic screening of ≈20, 000 exposed persons and subsequent treatment for those who tested positive (n = 90) with praziquantel†
November 27, 2014–March 25, 2015	Identification of 20 additional cases No case was acquired after exposure in 2014‡
May–September 2014	Malacologic investigation in 38 sites in Corsica (20 rivers) with identification of *Bulinus* truncatus snails in the Cavu, Solenzara, Tarcu, and Osu rivers (none was found infected with *Schistosoma* spp.)
Ongoing	Screening of cattle for *Schistosoma bovis* infection.

*Data sources (*1*–*4*).
†Of the 90 local cases, 20 had parasite eggs in urine samples, 24 had urinary or gynecologic symptoms that could be attributed to schistosomiasis. Half of the cases were in persons under 16 years of age, and many were familial clusters. Nineteen cases were in persons living in Corsica, 17 in residents of Provence-Alpes-Côte d'Azur region in southern mainland France, 54 in 13 other regions of mainland France.
‡Including 6 cases with parasite eggs in urine.

Technical Appendix Table 2. Tourism statistics for Corsica, France

	% of total tourists	
Country of origin	Corsica (May–September 2012), N = 2,700,000*	Porto-Vecchio (January–December 2011), N = 99,565†
Continental France	70.0	82.8
Italy	11.0	5.6
Belgium	5.9	1.0
Germany	5.0	3.4
Switzerland	5.0	0.5
United Kingdom	1.9	2.5
United States and Canada	Not documented	1.2
The Netherlands	Not documented	0.9
Spain	Not documented	0.7
Other	1.2	1.4

*Data source (*5*).
†Data source (*6*).

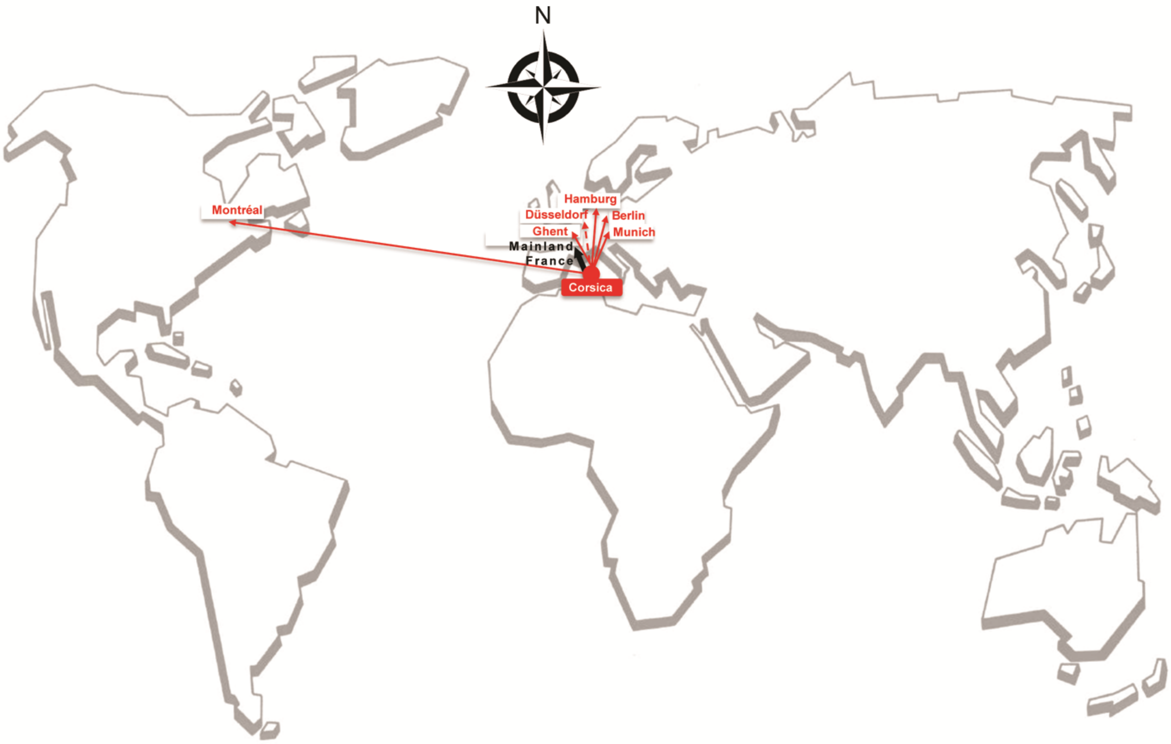

Technical Appendix Figure. Epidemiologic tracking for cases of schistosomiasis diagnosed during 2013–2014 in international travelers and French residents who bathed in various rivers in Corsica, France. Solid red arrows indicate cases identified through the GeoSentinel Surveillance Network and European Travel Network among international travelers (i.e., 11 patients described in this study [9 from Germany, 1 from Canada, 1 from Belgium]). Black arrow indicates cases detected by public health screening among French nationals from mainland France (91 cases). Dashed red arrow indicates international cases reported in the literature (5 from Germany).

References

1. Haut Conseil de la Santé Publique. Avis relatif au relatif au dépistage et au traitement des infections à *Schistosoma haematobium*. May 23, 2014 [cited 2015 Mar 22]. http://www.hcsp.fr/explore.cgi/avisrapportsdomaine?clefr=428

2. Ministère des Affaires Sociales et de la Santé. Communiqué de presse. Recommandations pour les personnes potentiellement exposées à la bilharziose après une baignade dans la rivière Cavu

(Corse du sud). June 16, 2014 [cited 2015 Mar 22]. http://www.sante.gouv.fr/IMG/pdf/CP_Bilharziose_16_juin_2014-3.pdf.

3. Institut National de Veille Sanitaire. Bilharziose urinaire: cas autochtones exposés au Cavu en Corse. Veille Hebdo, Provence-Alpes-Côte d'Azur/Corse. Point n°2014–48 publié le 28 novembre 2014 [cited 2015 Mar 3]. http://www.invs.sante.fr/fr/Publications-et-outils/Points-epidemiologiques/Tous-les-numeros/Sud/2014/Surveillance-epidemiologique-en-Paca-et-en-Corse.-Point-au-28-novembre-2014

4. Agence nationale de sécurité sanitaire de l'alimentation, de l'environnement et du travail. Avis relatif aux "critères de levée d'interdiction et d'autorisation de la baignade dans la rivière du Cavu (Corse du Sud) suite à la survenue de cas de bilharziose uro-génitale" [cited 2015 May 10]. https://www.anses.fr/fr/system/files/EAUX2015sa0036.pdf

5. Agence du Tourisme de la Corse A. Observation et stratégie touristiques 2013 [cited 2015 Mar 22]. http://www.bastia.aeroport.fr/Administration/accueil/pdf/pdfdossp20121227141029.pdf

6. Office Municipal de Tourisme Porto-Vecchio. Tableau de bord–année 2011 [cited 2015 Mar 22]. http://www.ot-portovecchio.com/userfiles/files/Stats/stats%202011.pdf
